# Protective effects and mechanisms of ellagic acid on intestinal injury in piglets infected with porcine epidemic diarrhea virus

**DOI:** 10.3389/fimmu.2024.1323866

**Published:** 2024-01-22

**Authors:** Zhuan Song, Cuifang Deng, Qinyin Chen, Shengnan Zhao, Peng Li, Tao Wu, Yongqing Hou, Dan Yi

**Affiliations:** ^1^ Hubei Key Laboratory of Animal Nutrition and Feed Science, Wuhan Polytechnic University, Wuhan, Hubei, China; ^2^ R&D Department, Hubei Horwath Biotechnology Co., Ltd, Xianning, Hubei, China

**Keywords:** ellagic acid, interferon, JAK2/STAT3 signaling, piglets, porcine epidemic diarrhea virus

## Abstract

The present study was conducted to decipher the protection effects of ellagic acid (EA) on piglets infected with porcine epidemic diarrhea virus (PEDV). Thirty 7-day-old piglets were randomly assigned to three treatment groups: control, PEDV, and EA + PEDV groups. After a 3-day period of adaption, piglets in the EA + PEDV group were orally administered with 20 mg/kg·BW EA during days 4-11 of the trial. On day 8, piglets were orally administered with PEDV at a dose of 10^6^ TCID_50_ (50% tissue culture infectious dose) per pig. Additionally, intestinal porcine epithelial (IPEC-1) cells infected with PEDV were used to investigate the anti-PEDV effect of EA *in vitro*. The results showed that EA at a dose of 10-40 μmol/L increased the viability of PEDV-infected IPEC-1 cells, and EA administration mitigated intestinal edema in piglets challenged with PEDV. Further studies indicated that EA treatment significantly increased the proportion of white blood cells in blood and concentrations of IL-6, IL-1β, and IL-10 in the serum, but decreased the TNF-α content and gene expression of *IL-6*, *IL-1β*, *TNF-α*, and *CXCL2* in the jejunum. Moreover, EA intervention considerably elevated the activity of total superoxide dismutase (T-SOD), but decreased the H_2_O_2_ concentration in the ileum of piglets. Importantly, EA suppressed the increased expression of antiviral-related genes and proteins (including MXI, ISG15, HSP70, and p-IRF7) induced by PEDV challenge in the jejunum. Furthermore, PEDV infection increased the protein abundance of p-JAK2 and p-STAT3, which were further enhanced by EA supplementation. In conclusion, our results revealed that EA could promote the restoration of intestinal homeostasis by regulating the interferon pathway that was interrelated with the activation of JAK2/STAT3 signaling. These findings provide theoretical basis for the use of EA as a therapy targeting PEDV infection in piglets.

## Introduction

Porcine epidemic diarrhea (PED), caused by porcine epidemic diarrhea virus (PEDV), is a highly pathogenic intestinal infectious disease in pigs. PEDV mainly infects and proliferates in villus enterocytes of the small intestine and it can lead to intestinal function destruction, absorption dysfunction, severe diarrhea, and even death of piglets (1). The emergence of porcine epidemic diarrhea was first reported in Britain in 1971 and then rapidly spread to other countries, which had an adverse impact on the pig industry (2, 3). At present, the effective prevention and control measures of PEDV infection mainly depend on vaccines. However, there are still some disadvantages, such as the enhancement of virulence and incomplete inactivation of traditional vaccines, which in turn results in the continuous recombination and mutation of PEDV and makes the prevention and control of PED more difficult (4). In the no-antibiotic era, it is of great practical significance to develop nutritional intervention strategies with anti-PEDV infection and protection of normal intestinal function.

Ellagic acid (EA) is a kind of polyphenol dinolactone, which exists widely in kinds of fruits and nuts and has multi-biological functions, such as anti-oxidation, anti-inflammation, antiviral and antibacterial activities (5). The four phenolic hydroxyl groups of EA are the basis for the reaction with various reactive oxygen species (ROS). The phenolic hydroxyl groups can provide H^+^, which can combinate with oxygen free radicals and reduce the content of oxygen free radicals in the body (6, 7). Studies have reported that EA possess the anti-inflammatory function by inhibiting the production of inflammatory cytokines and regulating the intestinal microbial structure (8, 9). Moreover, the antiviral activity of EA is associated with inhibiting virus multiplication, reducing virus titer and preventing virus from binding to host cell receptors. Importantly, it has been demonstrated that EA could improve intestinal health in weaned piglets by increasing gene expression of intestinal tight junction proteins and reducing mRNA levels for inflammatory cytokines (10). Based on the potential benefits of EA, we hypothesized that EA might also be advantageous to protect against PEDV-induced intestinal damage in piglets. Therefore, we conducted the present study to confirm this speculation and seek to reveal the relevant mechanisms by combining *in vitro* and *in vivo* experiments.

## Materials and methods

### Cell culture and viability detection

Intestinal porcine epithelial (IPEC-1) cells were cultured in DMEM/F-12 medium supplemented with 10% FBS, 1% penicillin-streptomycin, 1% insulin-transferrin-selenium, and 0.5% epidermal growth factor at 37°C with 5% CO_2_. 6×10^3^ cells per well were seeded in a 96-well plate. When cells fusion reached 40%, they were incubated with medium added with EA (0, 10, 20, 40, 60, 80 μmol/L) for 72 h. A cell counting kit-8 (Beyotime, Shanghai, China) was used to measure the cell viability according to the manufacturer’s instructions. Moreover, another 96-well plate was used to determine the effect of EA on the viability of PEDV-infected cells. When cells fusion reached 100%, IPEC-1 cells were infected with PEDV (MOI=0.1) for 1 h, then the cell supernatant was removed and cells were treated with EA (0, 2.5, 5.0, 10, 20, 40 μmol/L) for 24 h to detect the cell viability by the cell counting kit-8. The detailed timeline of these *in vitro* experiments was shown in **Supplementary Table 1**.

### Monolayer transepithelial electrical resistance determination

Cell monolayer transepithelial electrical resistance (TEER) was determined according to the method of Ji et al. (11). Briefly, IPEC-1 cells were seeded in the apical side of transwell inserts in 12-well plates with a density of 2 × 10^4^ cells per well. Cells were incubated with 10 μmol/L EA for 72 h. The TEER of cells was determined by using a Millicell ERS-2 Volt-Ohm Meter (Millipore, USA) on the indicated times and cells were collected for total protein extraction. In addition, IPEC-1 cells were seeded in the apical side of transwell inserts in another 12-well plates with a density of 2 × 10^6^ cells per well. Cells were challenged with PEDV for 1 h after 100% cell fusion. Then, the TEER of cells at different time points was measured. Once the minimum TEER approached 0, cells were collected to extract total RNA for the detection of *PEDV-M* and *PEDV-N* genes expression. The detailed timeline of the above experiments was shown in **Supplementary Table 1**.

### Animal experimental design

Thirty 7-day-old crossbred (Duroc × Landrace × Large White) healthy piglets were randomly assigned to three treatment groups: control, PEDV, and EA + PEDV groups. Each group contained 10 replicates with one pig per replicate. The entire experiment period was 11 days. The first three days were adaptation period and all piglets were fed with the liquid milk replacer. Nutrient components of the milk replacer were shown in **Table 1**. During days 4 to 10 of the trial, piglets in the EA + PEDV group were orally administered with 20 mg/kg·BW EA (purchased from Macklin Inc., Shanghai, China; purity ≥ 90%; dissolved in the liquid milk replacer), and piglets in the other two groups were treated with the same volume of milk replacer. On day 8, piglets in the PEDV and the EA + PEDV groups were orally received 1 mL PEDV at a dose of 10^6^ TCID_50_ (50% tissue culture infectious dose) per pig, while those in the control group consumed the same volume of sterile saline solution.

**Table 1:** Nutrient components of the milk replacer (as fed basis), 100%.

Items	Crude protein	Crude ash	Crude fiber	Moisture	Lysine	NaCl	Calcium	Total phosphorus
**Milk replacer**	≥20.0	≤9.0	≤1.0	≤10.0	≥1.4	0.3-1.5	0.4-1.1	≥0.3

Piglets were carefully observed daily throughout the trial period to record their diarrhea occurrence and health status. On day 11 of the trial, after overnight fasting, all piglets were orally administered with 10% D-xylose (1 mL/kg·BW) and anterior vena cava blood was collected 1 h later. Subsequently, all piglets were sacrificed to collect intestinal samples. Intestinal damage scores were evaluated as follows: 0: no visible intestinal or lung damage; 1: sporadic bleeding points in the intestines and lungs; 2: obvious damage in the intestine, lung, and liver can be seen by the naked eye, intestinal ulcers appear, and the intestinal wall becomes thin and permeable; 3: massive lung bleeding, intestinal ulcer, erosion, severe thinning of the intestinal wall and congestion. About 1-cm-long intestine segments were fixed in 4% paraformaldehyde to observe intestinal morphology, and the remaining intestines were rapidly frozen in liquid nitrogen and stored at -80°C until further analysis.

### Blood indices

The collected blood was placed at room temperature for 1 h, centrifuged at 3000 g, 4°C for 10 min, and the upper serum was collected. The concentration of D-xylose in the serum were measured using a commercially available kit (Jiancheng Institute of Biological Technology, Nanjing, China) according to the instruction. Blood cell counts were analyzed by the automated hematology analyzer (Siemens ADVIA 2120i, Germany) using the whole blood.

### Cytokine level detection

The contents of TNF-α, IL-1β, IL-6, IL-10, TGF-β1, and IFN-α in the serum were determined by using commercial ELISA kits (AAT Bioquest, California, CA, USA) according to the manufacturer’s guidelines. The jejunum tissue was accurately weighed and 9 times the volume of normal saline was added for mechanical homogenization under ice bath condition. The homogenate was centrifuged at 3000 g, 4°C for 10 min, and the supernatant was taken to measure jejunal TNF-α, IL-1β, IL-6, IgA, IL-10, and IFN-α concentrations also by using commercial ELISA kits (AAT Bioquest, California, CA, USA) according to the manufacturer’s guidelines.

### Antioxidant related indices in the jejunum and ileum

Homogenates in the jejunum and ileum were used to determine antioxidant-related indexes. Activities of total superoxide dismutase (T-SOD) and myeloperoxidase (MPO), as well as the concentration of hydrogen peroxide (H_2_O_2_) were calculated by the colorimetric method and standard curve according to commercially available kits (Nanjing Jiancheng Bioengineering Institute, Nanjing, China).

### Morphological structure analysis in the intestine

The morphological structure of small intestines was observed as previously described (12). Briefly, 4% paraformaldehyde fixed intestine samples were dehydrated and embedded in paraffin. Then, sections of 6-µm thickness were deparaffinized in xylene and dehydrated in ethanol for hematoxylin and eosin (H&E) staining. Villus height, crypt depth, and villus width in each section were quantitatively analyzed as described by Frankel et al. (13). Briefly, villi height is the vertical distance from the tip of the villi to the opening of the crypt; crypt depth is the vertical distance from the opening of the crypt to the base of the crypt; villus surface area is the product of villus height and villus width. 10 villi with the most complete morphological structure were selected, and the intestinal morphological structure of each sample was measured by the Olympus BX41 microscope (Olympus, Tokyo, Japan) with Image-Pro Plus 6.0 software (Media Cybernetics, Rockville, MD). Ratio of villus height to crypt depth and villus surface area were calculated and recorded.

### Total RNA extraction and quantitative real-time PCR

The RNAiso Plus (Takara, Dalian, China) reagent was used to extract total RNA. Sequentially, cDNA was synthesized using the PrimeScript^®^RT reagent kit with gDNA Eraser (Takara, Dalian, China). Real-time quantitative PCR (qRT-PCR) was performed by using the ABI 7500 real-time PCR system (ABI 7500, Alameda, CA, USA) with SYBR^®^ Premix Ex Taq™ (Tli RNaseHPlus) (Takara, Dalian, China). All the operation steps were completed according to the instruction of the manufacturer. Relative gene expression was determined using the 2^­ΔΔCt^ method and the *RPL4* gene was used for normalization. The primer sequences were shown in **Supplementary Table 2**.

### Western blot analysis

The protein of jejunum was extracted by a whole protein extraction kit (KeyGEN, Jiangsu, China), and the protein concentration was quantified by a BCA kit (Beyotime, Shanghai, China). Equal amounts of protein were separated by SDS-PAGE gels, followed by transferring onto PVDF membranes (Millipore, Billerica, MA, USA). The membrane bands were incubated with primary antibodies overnight at 4°C, and then incubated with secondary antibodies for 2 h at the room temperature. Finally, the grayscale value of protein band was determined by using an imaging system (Alpha Innotech FluorChem FC2, CA, USA). Antibodies used in the present study were as follows: MX1 (ab222856, Abcam, 1:1000), ISG15 (ab233071, Abcam, 1:1000), HSP70 (ADI-SPA-810-F, Enzo Life Sciences, 1:1000), IRF7 (QC8422, Sigma, 1:1000), p-IRF7 (PA5-114591, Invitrogen, 1:2000), JAK2 (#3230, Cell Signaling Technology, 1:2000), p-JAK2 (#3776, Cell Signaling Technology, 1:2000), STAT3 (#30835, Cell Signaling Technology, 1:1000), p-STAT3 (#9145, Cell Signaling Technology, 1:1000), Occludin (TC259714, Invitrogen, 1:2000), ZO-1 (61-7300, Invitrogen, 1:2000), Claudin-1 (RF217968, Invitrogen, 1:2000), E-cadherin (PA5-142828, Invitrogen, 1:2000), and β-actin (PA1–46296, Invitrogen, 1:4000).

### Statistical analysis

Data expressed as means ± SEM and all data were analyzed by one-way ANOVA or non-paired t test with SPSS 26.0 statistical software (SPSS, Inc., Chicago, IL, USA). The *Duncan* multiple comparison method was used to determine the differences between means among the treatment groups. Graphs were created by using the GraphPad Prism 8.0 software (GraphPad Software, Inc., San Diego, CA, USA). *P* < 0.05 was considered significantly different between groups.

## Results

### EA promoted the viability and barrier function in PEDV-infected IPEC-1 cells

**Figure 1:**
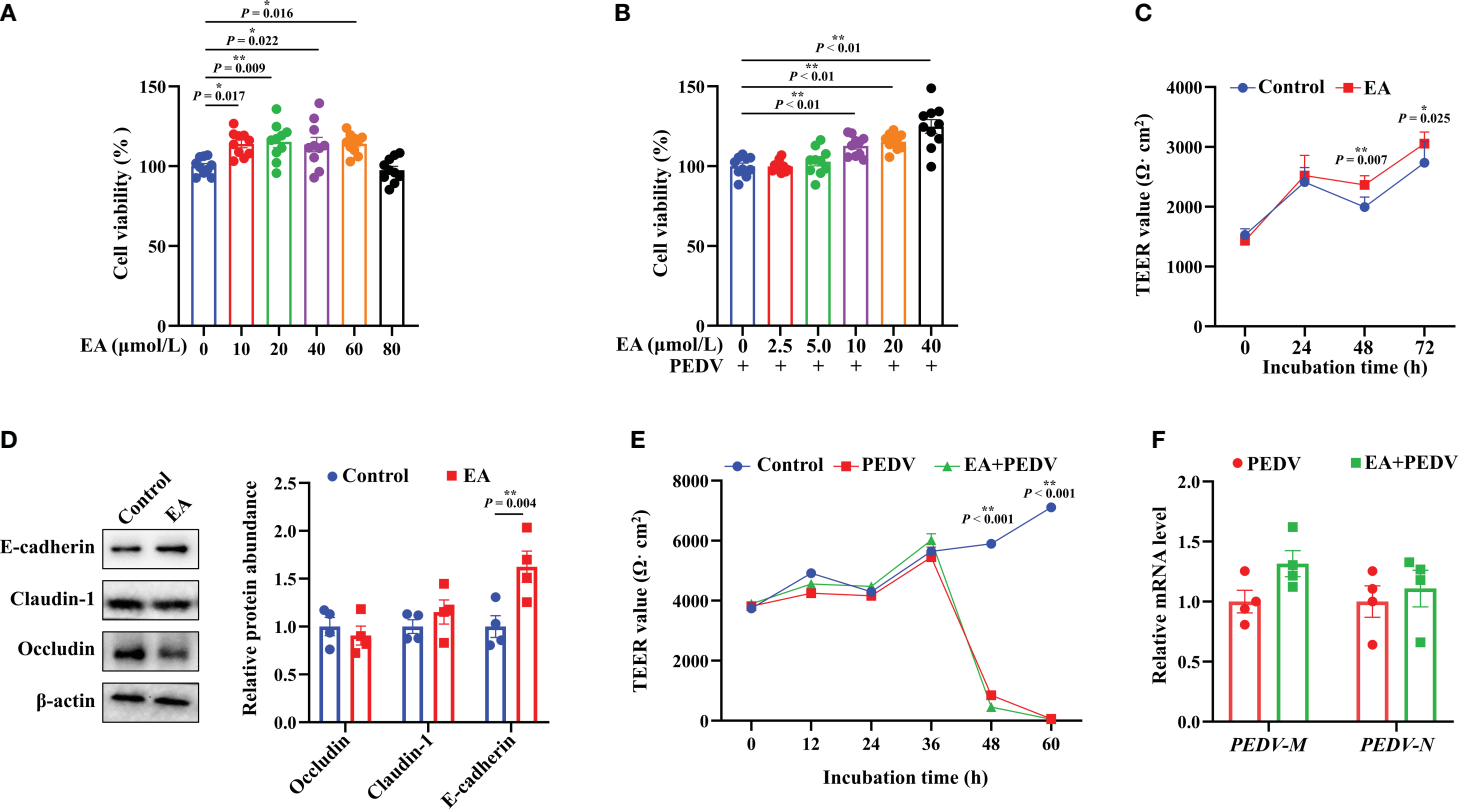
Effects of EA treatment on the viability and barrier function of IPEC-1 cells infected with PEDV. **(A)** Concentration-dependent effects of EA on the proliferation of IPEC-1 cells, n = 10; **(B)** Alleviation of EA on the cytotoxicity in PEDV-infected IPEC-1 cells, n = 10; **(C)** TEER values of EA treated-cells, n = 8; **(D)** Expression levels of tight junction proteins at 72 h, n = 4; **(E)** Effect of EA on the TEER values in PEDV-infected cells, n = 4; **(F)** mRNA levels for PEDV marker genes, n = 4. Data are presented as means ± SEMs. *P < 0.05, **P < 0.01.

Results showed that 10-60 μmol/L EA significantly promoted the proliferation of IPEC-1 cells by 13.57-15.26%, but the dose of 80 μmol/L had no significant effect (**Figure 1A**, *P* < 0.05). Moreover, 10-40 μmol/L EA also noticeably improved the viability of PEDV-infected cells by 12.68-24.03% (**Figure 1B**, *P* < 0.05). Based on these results, therefore, we chose 10 μmol/L EA in the following studies. Interestingly, cells incubated with 10 μmol/L EA exhibited higher TEER value than those in the control group at 48 h without PEDV infection, which suggested that EA had the ability to reinforce the barrier function of IPEC-1 cells (**Figure 1C**, *P* < 0.05). Moreover, EA treatment notably increased the protein expression of E-cadherin at 48 h (**Figure 1D**, *P* < 0.05). Nevertheless, EA supplementation didn’t affect the membrane resistance of PEDV-infected cells (**Figure 1E**). The N and M gene encodes the nuclear protein and the membrane protein of PEDV, respectively, both of which are marker genes for the detection of PEDV viral load. In the current study, we found that EA failed to inhibit the replication of PEDV *in vitro*, which was reflected by no obvious changes in the mRNA expression of *PEDV-N* and *PEDV-M* genes (**Figure 1F**).

### Effect of EA on the intestinal injury of PEDV-infected piglets

**Figure 2:**
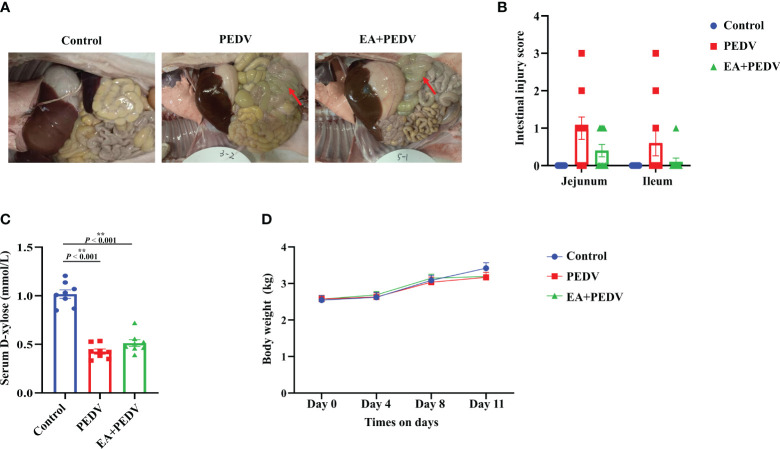
Effect of EA administration on the intestinal injury and body weight change in piglets infected with PEDV. **(A)** Representative pictures showing the intestinal edema; **(B)** Intestinal injury scores of the jejunum and ileum; **(C)** D-xylose concentration in the serum; **(D)** Body weight changes. Data are presented as means ± SEMs (n = 10). *P < 0.05, **P < 0.01. Red arrows show intestinal edema.

Compared with the control group, anatomical observation showed that the intestinal wall of PEDV-infected piglets was thinner and intestinal flatulence was exerted (**Figure 2A**). Although the statistical difference was not significant, supplementation with EA reduced intestinal edema and intestinal injury scores (**Figure 2B**). Plasma D-xylose concentration is an important indicator for intestinal absorption function. PEDV infection strongly decreased D-xylose concentration compared with the control group, and EA intervention had no significant influence on D-xylose level in the serum of piglets (**Figure 2C**). Additionally, EA had no effect on body weight in piglets (**Figure 2D**).

### Effects of EA on the intestinal morphology of PEDV-infected piglets

**Figure 3:**
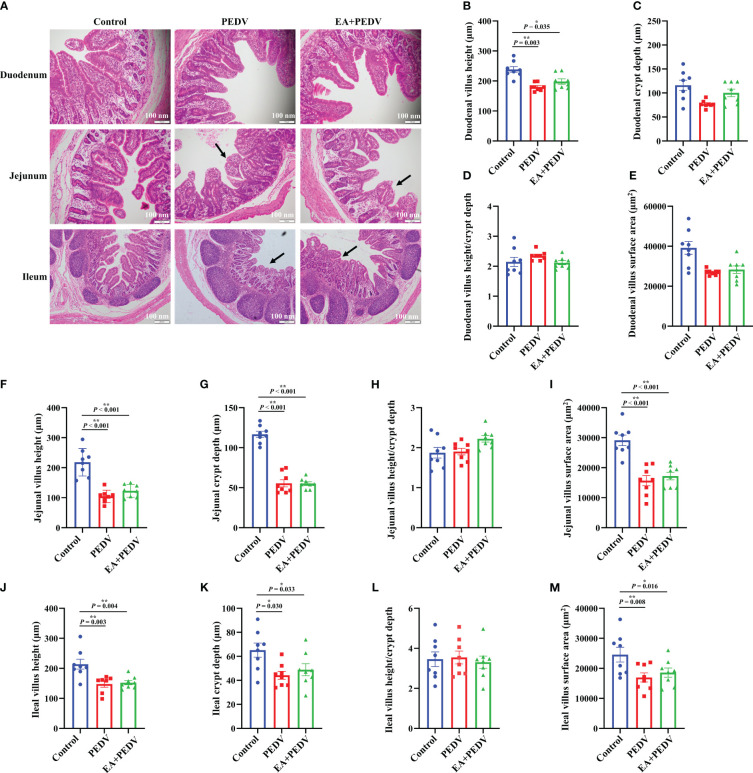
Effects of EA administration on the villus structure in piglets infected with PEDV. **(A)** H&E staining. Scale bar: 100 nm; Villus height **(B, F, J)**, crypt depth **(C, G, K)**, villus height/crypt depth **(D, H, L)**, and villus surface area **(E, I, M)**. Data are presented as means ± SEMs (n = 10). *P < 0.05, **P < 0.01. Black arrows show the atrophy or shedding of intestinal villi.

As presented in **Figure 3A**, intestinal villi were severely atrophied or deciduous due to PEDV infection, EA had no obvious influence on the villus morphology. Statistical results showed that PEDV infection significantly decreased villus height in all small intestines, as well as crypt depth, and villus surface area in the jejunum and ileum (*P* < 0.05); EA treatment neither restored the intestinal villi height nor crypt depth to the normal value (**Figures 3B–M**). These results indicated that EA could not alleviate the damage of intestinal villus caused by PEDV infection.

### Inflammation-related indicators in blood and the jejunum

**Figure 4:**
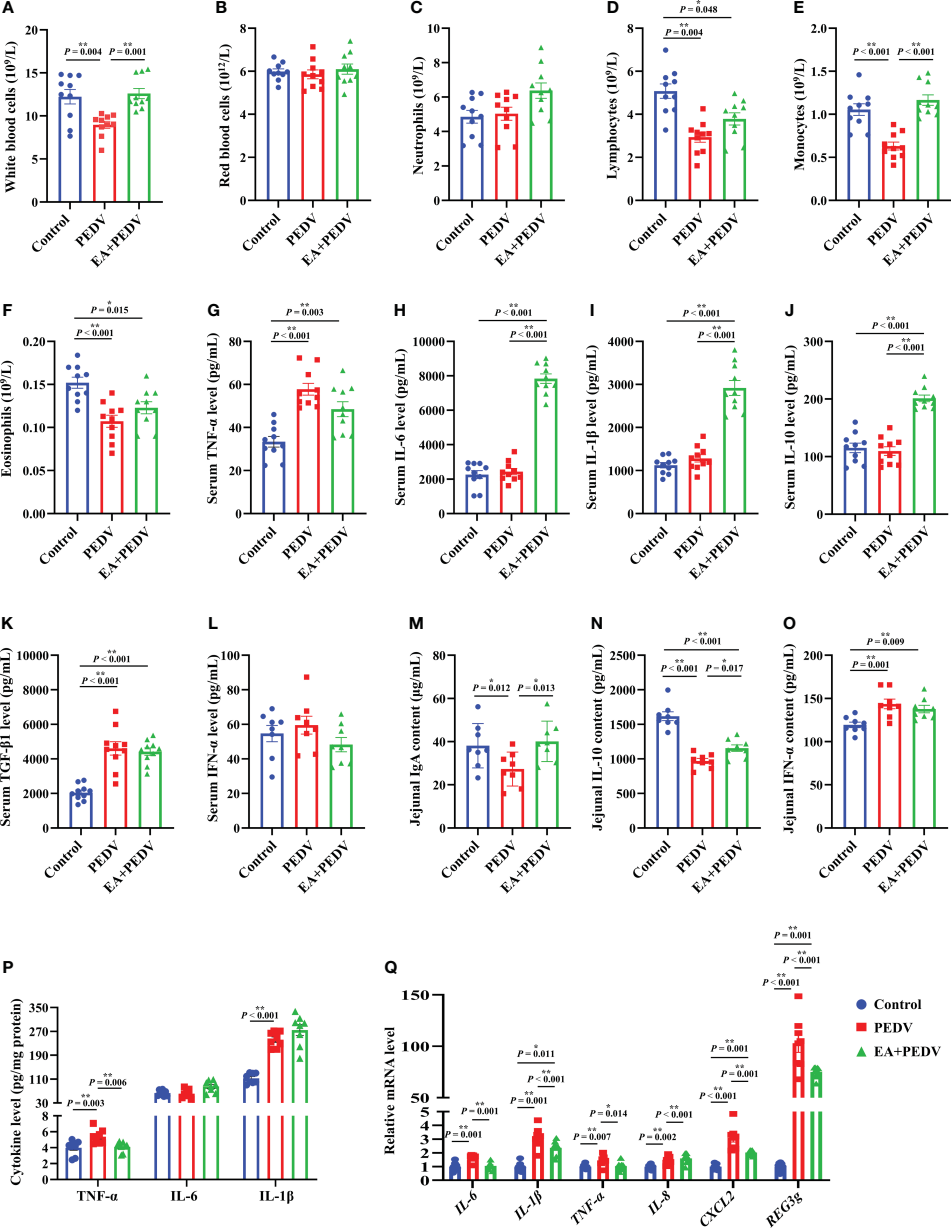
Effects of EA administration on inflammatory indices of blood and the jejunum in piglets infected with PEDV. **(A–F)** Blood cell counts; **(G–L)** Concentrations of TNF-α, IL-6, IL-1β, IL-10, TGF-β1, and IFN-α in the serum; **(M–P)** Concentrations of IgA, IFN-α, TNF-α, IL-6, and IL-1β in the jejunum; **(Q)** mRNA levels for inflammation-related genes in the jejunum. Data are presented as means ± SEMs (n = 8). *P < 0.05, **P < 0.01. TNF-α, tumor necrosis factor α; IL, interleukin; TGF-β1, transforming growth factor β1; CXCL2, chemokine ligand 2; REG3g, regenerating islet-derived 3 gamma.

As displayed in **Figures 4A–F**, PEDV infection significantly decreased the proportion of white blood cells, lymphocytes, monocytes, and eosinophils in comparison with the control group (*P* < 0.05), and EA supplementation conversely increased the number of white blood cells and monocytes. Additionally, compared with the control group, PEDV infection considerably increased the serum concentrations of TNF-α and TGF-β1 (**Figures 4G, K**), and EA treatment decreased the TNF-α level (*P* < 0.05). PEDV infection had no significant effect on IL-6, IL-1β, IL-10, and IFN-α levels in the serum (**Figures 4H–J, L**), while EA administration highly elevated concentrations of IL-6, IL-1β, and IL-10 when compared with PEDV group (*P* < 0.05).

We also examined the relevant indices of jejunal inflammatory response. As shown in **Figures 4M, N**, EA significantly reversed the decrease of IgA and IL-10 contents in the jejunum of piglets induced by PEDV infection (*P* < 0.05). Additionally, concentrations of IFN-α, TNF-α, and IL-1β in the jejunum were enhanced by PEDV challenge, whereas EA treatment decreased the TNF-α level (**Figures 4O, P**, *P* < 0.05). In line with the results regarding cytokine content, we also found that the mRNA levels for *IL-6*, *IL-1β*, *TNF-α*, *IL-8*, chemokine ligand 2 (*CXCL2*), as well as regenerating islet-derived 3 gamma (*REG3g*) were more abundant in the PEDV group than those in the control group, and these gene expressions were blunted by EA receiving (**Figure 4Q**, *P* < 0.05), except for *IL-8*. These results emphasized that EA can ameliorate the inflammatory response of PEDV-infected piglets.

### Effects of EA on the intestinal antioxidant function of PEDV-infected piglets

**Figure 5:**
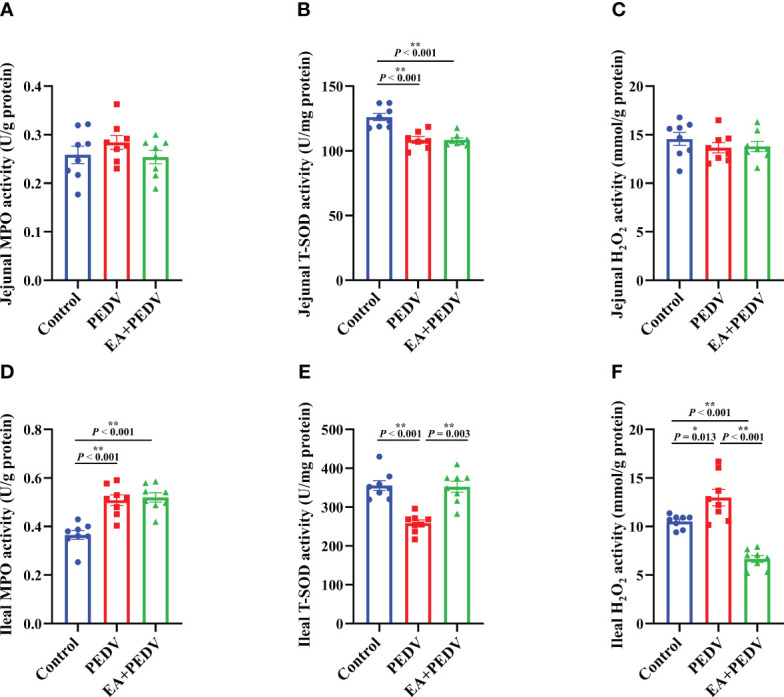
Effects of EA administration on the antioxidation function in piglets infected with PEDV. Activities of MPO **(A, D)** and T-SOD **(B, E)**, as well as the concentration of H_2_O_2_ **(C, F)**. Data are presented as means ± SEMs (n = 8). *P < 0.05, **P < 0.01. MPO, myeloperoxidase; T-SOD, total superoxide dismutase; H_2_O_2_, hydrogen peroxide.

The antioxidant enzymes and related products of the jejunum and ileum were determined to investigate the antioxidative effect of EA on piglets. Compared with the control group, PEDV infection considerably reduced the activity of T-SOD both in the jejunum and ileum (**Figures 5B, E**, *P* < 0.05), had no effect on jejunal MPO and H_2_O_2_ levels (**Figures 5A, C**) but increased the activity of MPO and the concentration of H_2_O_2_ in the ileum (**Figures 5D, F**, *P* < 0.05). EA intervention obviously restored the T-SOD activity and the H_2_O_2_ content to the normal level in the ileum of pigs (*P* < 0.05).

### The antiviral effect of EA in the jejunum of PEDV-infected piglets

**Figure 6:**
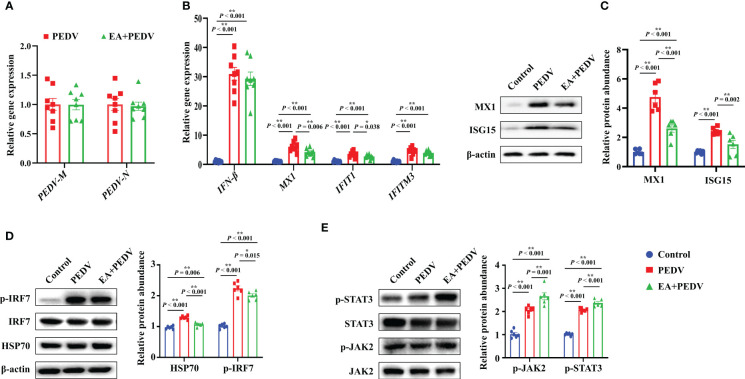
Expression of antiviral-related genes and proteins in the jejunum. **(A)** mRNA levels of PEDV marker genes; **(B)** mRNA level for genes involved in the interferon pathway; Protein abundance of MX1 and ISG15 **(C)**, HSP70 and p-IRF7 **(D)**, p-JAK2 and p-STAT3 **(E)**. Data are presented as means ± SEMs (n = 8). *P < 0.05, **P < 0.01. IFN, interferon β; MX1, myxovirus resistant 1; IFIT1, interferon-induced protein with tetratricopeptide repeats 1; IFITM3, interferon induced trans-membrane proteins 3; ISG15, interferon-stimulated gene 15; HSP70, heat shock protein 70; IRF7, interferon regulatory factor 7; JAK2, Janus tyrosine kinase 2; STAT3, signal transducer and activator of transcription 3.

Consistent with the results *in vitro*, no significant differences were observed in *PEDV-M* and *PEDV-N* mRNA levels between the PEDV and EA groups in the jejunum of piglets (**Figure 6A**). Nevertheless, mRNA levels for antiviral-related genes, such as interferon β (*IFN-β*), myxovirus resistant 1(*MX1*), interferon-induced protein with tetratricopeptide repeats 1 (*IFIT1*), and interferon induced trans-membrane proteins 3 (*IFITM3*), were generally increased by PEDV infection. Among these genes, EA consumption lowered the *MX1* and *IFIT1* expressions (**Figure 6B**, *P* < 0.05). Moreover, PEDV infection significantly upregulated the protein abundance of MX1, interferon-stimulated gene 15 (ISG15), heat shock protein 70 (HSP70), and the phosphorylation of interferon regulatory factor 7 (p-IRF7), which was inversely changed by EA supplementation (**Figures 6C, D**, *P* < 0.05). Further exploration showed that both the protein abundance of p-JAK2 and p-STAT3 were elevated by PEDV challenge, and the addition of EA ulteriorly promoted the expression of p-JAK2 and p-STAT3 (**Figure 6E**, *P* < 0.05). These results indicated that although EA cannot inhibit PEDV proliferation, it had a certain antiviral potential through immunomodulatory effects.

### Effects of EA on jejunal nutrient transport function and barrier function in PEDV-infected piglets

**Figure 7:**
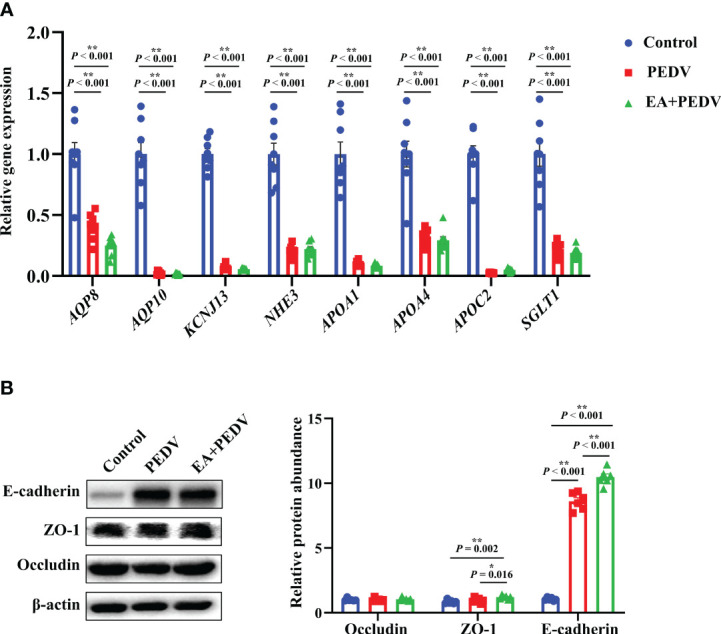
Expression of nutrient absorption and transport-related genes **(A)** and tight junction proteins **(B)** in the jejunum. Data are presented as means ± SEMs (n = 8). *P < 0.05, **P < 0.01. AQP, aquaporin; KCNL13, potassium inwardly-rectifying channel, subfamily J, member 13; NHE3, Na^+^/H^+^ exchangers; Apo, apolipoproteins; SGLT1, sodium/glucose cotransporter 1; ZO-1, zonula occludens-1.

As shown in **Figure 7A**, compared with the control group, PEDV infection decreased the relative mRNA abundance of ion channel-related proteins, mainly aquaporin (AQP8 and AQP10), potassium inwardly-rectifying channel, subfamily J, member 13 (KCNJ13), Na^+^/H^+^ exchangers (NHE3), and lipid transporter such as apolipoproteins (APOA1, APOA4, APOC2) and sodium/glucose cotransporter (SGLT1) (*P* < 0.05), but no apparent differences were observed in the expression of these genes between the PEDV and EA supplementation groups. Results about the expression of jejunal tight junction proteins showed that EA administration elevated the protein abundance of ZO-1 and E-cadherin in comparison with the PEDV group (**Figure 7B**, *P* < 0.05).

## Discussion

Lethal watery diarrhea in piglets, especially in weaned piglets, is still a popular problem in the world and is quite challenging. The continuous variation of virulent strains makes classical vaccines fail to provide effective protection against PEDV infection. It has been proved that EA is a promising feed additive with anti-oxidation, anti-inflammation, and growth-promoting properties. In the present study, we found that EA enhance enterocytes proliferation and barrier function, and prevented PEDV infection-induced intestinal inflammation and oxidative stress. This beneficial effect may be interrelated with the downregulation of inflammatory cytokines and antiviral-related genes, as well as the activation of JAK2/STAT3 signaling.

PEDV mainly proliferates in the small intestine, therefore, the PEDV-challenged IPEC-1 cells model was used to preliminarily evaluate whether EA has a protective effect against PEDV-infection. Our results showed that although EA could not inhibit the replication of PEDV, it enhanced the proliferative activity of PEDV-infected cells and increased the protein expression of E-cadherin and TEER value *in vitro*. E-cadherin is an intercellular adhesion protein that is generally distributed in the junctions between cells on the surface of epithelial cells (14). The enhanced E-cadherin expression suggested that EA may have the capability to improve intestinal barrier function in piglets. Thereafter, we conducted the animal experiment to further explore the protective effects of EA on intestine function of PEDV-infected piglets.

Given that addition of EA alone had no negative effect on the growth performance of piglets (**Supplementary Figure 1**), we focused on the regulatory effects of EA on the intestine function by using an experimental model of pigs infected with PEDV. In the present study, PEDV infection severely damaged the villus structure of small intestine, and thereby led to intestinal edema, intestinal wall thinning, and diarrhea. EA supplementation alleviated jejunal edema to some extent, but failed to improve the morphological integrity of villus. Intriguingly, Lu et al. found that dietary EA increased the average daily gain and reduce the diarrhea rate in weaned piglets (15). The reason for this inconsistent result may be due to the pigs with different physiological stages have different bioavailability of EA.

It is an important role for blood cell indicators to reflect the status of health and the capacity of body metabolism. Blood cells are divided into red blood cells, white blood cells and platelets (16). Among that, white blood cells are exudative and chemotactic, and their main functions are to phagocyte pathogens and secrete interferon and interleukin mediators. There are five types of white blood cells: neutrophils, eosinophils, basophils, lymphocytes and monocytes, and different cell subsets have their own special roles (16, 17). Viral infections usually impair the function of white blood cells, resulting in a decrease in the number of white blood cells, especially lymphocytes. Importantly, study found that there were significant neutropenia and lymphocytopenia in peripheral blood of pigs 2-3 days after African swine fever virus infection (18). EA treatment noticeably increased the counts of total white blood cells, and monocytes, which argued that EA can alleviate the immunosuppression caused by PEDV and improve the system immunity of piglets. Consistent with the increase in white blood cell count, EA intervention numerically elevated IL-6, IL-1β, and IL-10 levels in the serum. Note that the body has a function of self-immune regulation and inflammatory mediator are not continuously secreted. Serum cytokine contents in PEDV-infected piglets may have recovered when we sampled at the end of the trial. EA has an immunological intervention effect, which might cause a slower reduction of cytokine concentrations. Therefore, the capacity of self-maintaining homeostasis may ultimately result in higher IL-6, IL-1β, and IL-10 contents in the EA treatment group than in the PEDV infection group. Generally, these results revealed that EA may contribute to alleviating the inflammatory response in piglets induced by PEDV.

Complying with our previous studies (19, 20), a severe inflammatory response was triggered in the jejunum of PEDV-infected piglets, as characterized by the markedly decrease in IgA and IL-10 contents and increase in concentrations of IFN-α,TNF-α, and IL-1β in the jejunum, whereas EA addition conversely increased IgA and IL-10 concentrations and decreased the TNF-α level. Consistently, the increased gene expression of jejunal cytokines (*IL-6*, *IL-1β*, *TNF-α* and *CXCL2*) was also counteracted by EA intervention, which revealed that EA could ameliorate jejunal inflammation induced by PEDV infection. Proteins in the regenerating gene (REG) family have been found to serve as multifunctional molecules with antimicrobial, anti-apoptotic, anti-inflammatory, and probably immuno-regulatory effects (21). Accumulating evidence have uncovered the potential role of the REG3g in the development of inflammation-associated gastrointestinal diseases (22, 23). Our results presented that the relative gene abundance of *REG3g* was lower in EA treated piglets than those in the PEDV-challenged group. In line with our findings, Fan et al. also reported that PEDV infection was accompanied by upregulated *REG3g* expression in the jejunum (23), which highlighted that the REG3g may contribute essential functions for suppressing the PEDV replication.

Usually, the oxidation and antioxidant systems maintain a dynamic balance to prevent against the accumulation of ROS in the body. However, insufficient expression of antioxidant enzymes can result in incomplete clearance of ROS and causing oxidative stress (18). MPO is a specific marker in neutrophils and its elevation indicates a high proportion of neutrophils, which indirectly reflects the inflammatory response (24). SOD is an important antioxidant enzyme that mainly exists in the cytoplasm and it can facilitate the breakdown of superoxide radical into O_2_ or H_2_O_2_ to reduce oxidative stress. Total SOD (T-SOD) includes CuZn-SOD and Mn-SOD. In addition, as an oxidative metabolite, high concentration of H_2_O_2_ can diminish the integrity of cell membranes and then precipitate cell apoptosis (25). The physiological structure and function of the intestine make it constantly expose to the external environment, food oxides, bacteria and virus. Therefore, the intestine is more likely to produce excessive ROS, evoke intestinal inflammation and the damage of intestinal barrier function. In the present study, the activity of ileal MPO and H_2_O_2_ concentration were increased by PEDV challenge, while the T-SOD level was strongly lowered both in the jejunum and ileum. The changes in these indicators suggested that PEDV induced oxidative stress in the intestine of piglets, which was in concordance with our previous findings that the activity of antioxidant enzymes could be suppressed by PEDV infection (26). However, EA treatment considerably enhanced the activity of T-SOD, but decreased H_2_O_2_ concentration in the ileum, which indicated that EA could effectively alleviate oxidative damage in PEDV-infected piglets. Consistent with our findings, Xiao et al. reported that EA relieved paraquat-induced intestinal oxidative stress in weaned piglets (19). Collectively, EA might be a potential additive to prevent oxidative stress-mediated gut diseases.

Existing studies reported that EA possess a potential to inhibit the replication of HIV and SARS-CoV-2 virus *in vitro* (27–29). Considering that EA ameliorated the oxidative stress and inflammation elicited by PEDV infection in piglets, we proposed that EA had an anti-PEDV effect *in vivo*. However, concordant with results in IPEC-1 cells, EA also had no significant influence in mRNA levels for *PEDV-M* and *PEDV-N*, which suggested that EA failed to block the proliferation of PEDV. The body mainly relies on the immune system to fight viruses. After virus invasion, the body’s self-protection mechanism will be activated, resulting in increased expression of antiviral-related genes. Expression of genes (mainly *IFN-β*, *MX1*, *IFIT1* and *IFITM3*) involved in antiviral was generally increased in the jejunum of PEDV-infected piglets, and EA supplementation inversely blunted the transcriptional level of *MX1* and *IFIT1*. Importantly, EA treatment also decreased the protein abundance of MX1 and ISG15 in the jejunum. These results again suggested that EA can respond to PEDV infection through immunomodulatory effects. IFN-β is one kind of type I interferon that responses mainly by inducing the expression of IFN stimulate gene (ISGs) and producing a variety of antiviral factors, which not only has a direct antiviral effect, but also has a certain immune enhancement function (30). IFIT1 is a member of the interferon-induced protein with tetratricopeptide repeats family (IFITs) and it has extensive antiviral activity and anti-inflammatory effect. Genes in the IFITs family belong to ISG genes and virus infection can make the expression of them rapidly increased within a short time (31). Additionally, MX1 is also a member of ISGs, which recognizes the nucleocapsid structure of the virus and interferes with the invasion of viral nucleic acid fragments into cells (32). The alterations of these genes and protein expressions suggested that although EA treatment did not directly reduce PEDV proliferation, it conveyed a protection on the immune homeostasis through downregulating these ISG genes and proteins in the gut of PEDV-infected piglets. This phenomenon was consistent with our previously described that nutritional intervention can relieve intestinal damage induced by PEDV through the interferon signaling (26, 33).

Interferon regulatory factors (IRFs) are multifunctional transcription factors, which are critical for the production of type I IFN in regulating cell signal transduction and immune response. Thirty years ago, the JAK/STAT pathway was found to be involved in cells response to IFN (34). The secreted IFN activates JAK-STAT pathway according for inducting ISGs (35). IRFs comprise nine members, ranging from IRF1 to IRF9. Studies showed that IRF7 is mainly expressed in lymphocytes, splenocytes, thymocytes, dendritic cells and other immune-related cells (36). In the present study, we found that PEDV challenge increased the protein abundance of p-IRF7, p-JAK2 and p-STAT3, which was partly agreement with Li et al., who reported that virus in piglets can induce the transcription of type I interferon and stimulate the activation of JAK/STAT pathway by regulating key signaling molecules such as IRF7 and then trigger the innate immunity (37). Interestingly, EA treatment decreased the phosphorylation of IRF7, but ulteriorly enhanced the protein richness of p-JAK2 and p-STAT3. The JAK/STAT pathway is central to extracellular cytokine activated receptor-mediated signal transduction, which is involved in cell proliferation and differentiation, organ development, and immune homeostasis (34). We hypothesized that the addition of EA promoted the expression of other cytokines or growth factors, which could also activate the JAK2/STAT3 pathway. Additionally, our previous study found that puerarin, another plant extract, exerted anti-PEDV effects by activating STAT1 (38). Therefore, there may be common and specific mechanisms for the regulatory effects of plant extracts on activating the JAK/STAT pathway in anti-PEDV virus, which deserves further investigation. Moreover, as Park et al. described that HSP70 enhanced PEDV replication by interacting with membrane proteins (39). We also found that PEDV infection upregulated the HSP70 protein expression, which was restored by EA ingestion. Heat shock proteins (HSPs) are a class of chaperones that are responsible for maintaining cellular proteostasis and metabolism homeostasis (40). Recently, Lubkowska et al. summarized that HSP70 is essential for the proliferate of a wide range of virus, such as dengue virus, influenza A virus, human enterovirus, and the hepatitis C virus (41). Therefore, our study and Park et al. provided evidence that the HSP70 is of utmost importance for PEDV replication, and EA may be an effective additive to target HSP70 for improving immune response in PEDV-infected piglets.

Diarrhea is associated with abnormal expression of ion channel proteins that influence the digestion and absorption of nutrients. The gut is the main place for the digestion and absorption of nutrients, and ion channels play important role in nutrient transport and absorption. AQPs are water channels and are responsible for transporting the water from lumen to enterocytes (42); KCNJ13 is a member of the inwardly rectifying potassium channel family of proteins and accounting for potassium ions passing into a cell (43); NHE3 is mainly related to the exchange of sodium ions inside and outside the cell (44); APOA1, APOA4, and APOC2 are associated with lipid digestion and absorption in the small intestine (45); SGLT1 is responsible for glucose transport (46). In the present study, EA had no significantly influence on mRNA levels of ion channel-related and nutrient absorption-related genes, which may suggest that it did not promote the nutrient absorption of PEDV-infected piglets. This result may partially provide the explanation for the findings that EA could not exert the positive effects on the intestine morphology and body weight in PEDV-infected piglets.

Generally, the invasion of pathogens damages the integrity of the intestinal epithelium and decreases the expression of tight junction proteins. However, our results showed that PEDV infection increased the protein expression of E-cadherin. E-cadherin is a class of Ca^2+^-dependent intercellular adhesion molecules expressed in mammalian epithelial cells and it is essential for the maintenance of epithelial tissue formation, organ morphological development, and control of epithelial cell proliferation. The rise in E-cadherin expression is also associated with a recovery in the number of healthy epithelial cells (14, 47). Our seemingly contradictory result may be due to the body’s self-regulation ability to resist pathogen invasion under certain conditions by promoting the expression of proteins related to the intestinal barrier function. Moreover, partly consistent with results of *in vitro* experiments, EA administration further enhanced the protein abundance of ZO-1 and E-cadherin in the jejunum of PEDV-infected piglets, which indicated that the function of intestinal epithelium was improved by EA treatment and the increased E-cadherin may be contributing to fighting against the invasion of PEDV for piglets. Qin et al. also found that dietary EA supplementation attenuated intestinal damage with the enhancement of tight junction proteins in weanling piglets (8). Therefore, the alleviation of intestinal inflammatory response and oxidative stress by EA administration in PEDV-infected piglets may be benefit from its improving effect on intestinal barrier function.

## Conclusions

**Figure 8:**
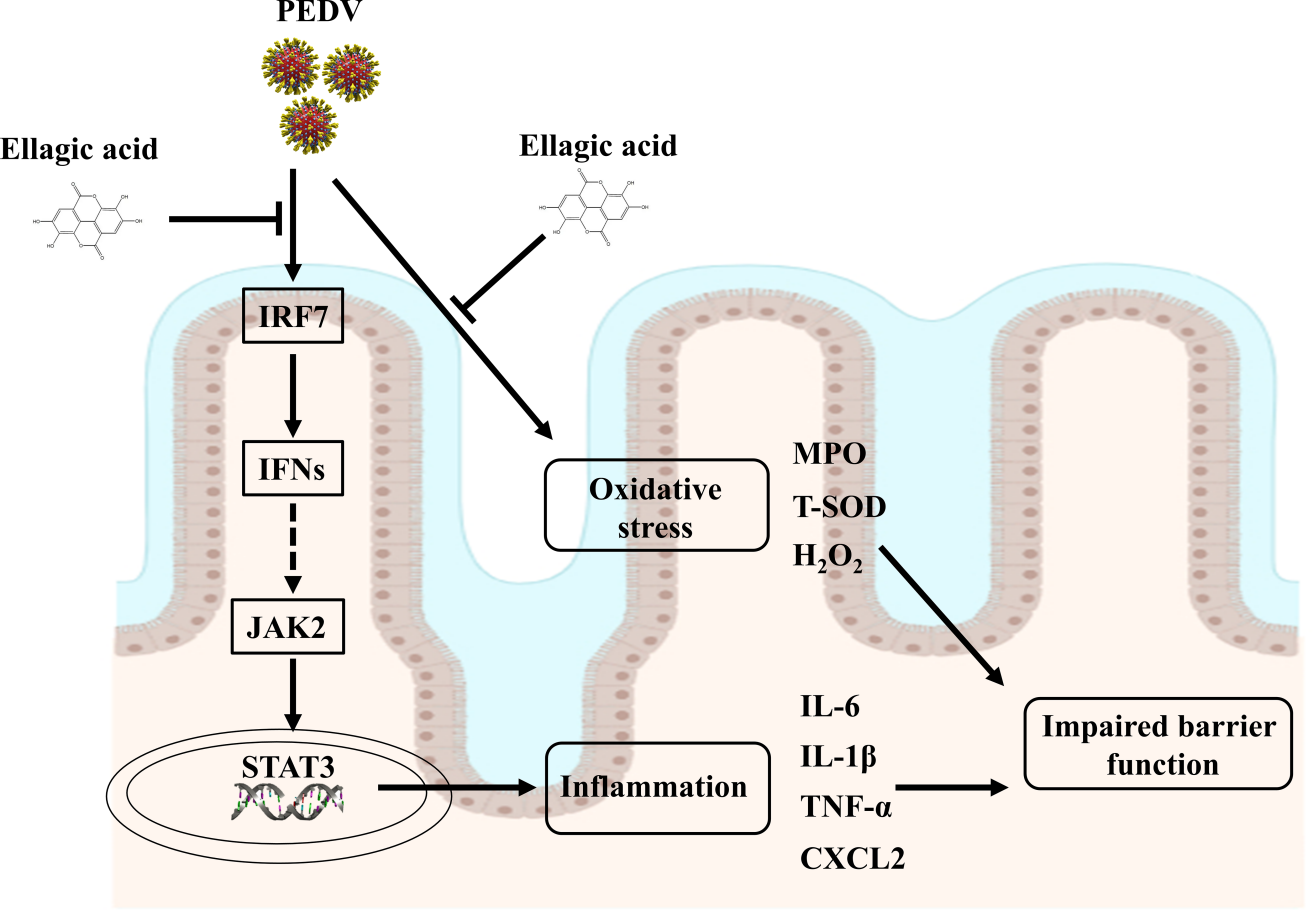
Suggested mechanisms of EA action on ameliorated intestinal injury in PEDV-infected piglets.

EA administration could alleviate oxidative stress and intestinal inflammation in PEDV-infected piglets, as manifested by increased T-SOD activity and decreased cytokine expressions. Furthermore, EA administration improved the antiviral function to restore intestinal homeostasis probably by regulating the interferon pathway and that was companied with the trigger of JAK2/STAT3 signaling (**Figure 8**). Overall, EA may function as a nutritional feed additive to protect against intestinal injury.

## Data availability statement

The datasets presented in this study can be found in online repositories. The names of the repository/repositories and accession number(s) can be found in the article/**Supplementary Material**.

## Ethics statement

The animal study was approved by the Animal Care and Use Committee of Wuhan Polytechnic University. The study was conducted in accordance with the local legislation and institutional requirements.

## Author contributions

ZS: Data curation, Formal analysis, Investigation, Writing – original draft. CD: Data curation, Investigation, Methodology, Writing – review & editing. QC: Investigation, Methodology, Writing – review & editing. SZ: Data curation, Investigation, Methodology, Writing – review & editing. PL: Formal analysis, Methodology, Software, Writing – review & editing. TW: Data curation, Methodology, Writing – review & editing. YH: Funding acquisition, Supervision, Conceptualization, Writing – review & editing. DY: Conceptualization, Funding acquisition, Writing – original draft, Writing – review & editing, Project administration, Supervision.

## Glossary

Apo: apolipoproteins
AQP: aquaporin
CXCL2: chemokine ligand 2
EA: ellagic acid
H&E: hematoxylin and eosin
H_2_O_2_: hydrogen peroxide
HSP70: heat shock protein 70
IFIT1: interferon-induced protein with tetratricopeptide repeats 1
IFITM3: interferon induced trans-membrane proteins 3
IFN: interferon β
IL: interleukin
IRF7: interferon regulatory factor 7
ISG15: interferon-stimulated gene 15
JAK2: Janus tyrosine kinase 2
KCNL13: potassium inwardly-rectifying channel
subfamily J: member 13
MPO: myeloperoxidase
MX1: myxovirus resistant 1
NHE3: Na^+^/H^+^ exchangers
PED: porcine epidemic diarrhea
PEDV: porcine epidemic diarrhea virus
REG3g: regenerating islet-derived 3 gamma
SGLT1: sodium/glucose cotransporter 1
STAT3: signal transducer and activator of transcription 3
TEER: transepithelial electrical resistance
TGF-β1: transforming growth factor β1
TNF-α: tumor necrosis factor α
T-SOD: total superoxide dismutase
ZO-1: zonula occludens-1
