# Supplementary material for: Protective effects and mechanisms of ellagic acid on intestinal injury in piglets infected with porcine epidemic diarrhea virus
Source: Front Immunol. 2024 Jan 22;15:1323866. doi: 10.3389/fimmu.2024.1323866 (PMC10845347; doi:10.3389/fimmu.2024.1323866)
Supplement: Supplementary file 1 [file DataSheet_1.docx]

Protective Effects and Mechanisms of Ellagic Acid on Intestinal Injury in Piglets Infected with Porcine Epidemic Diarrhea Virus

Supplemental Table 1. The detailed timeline of *in vitro* experiments


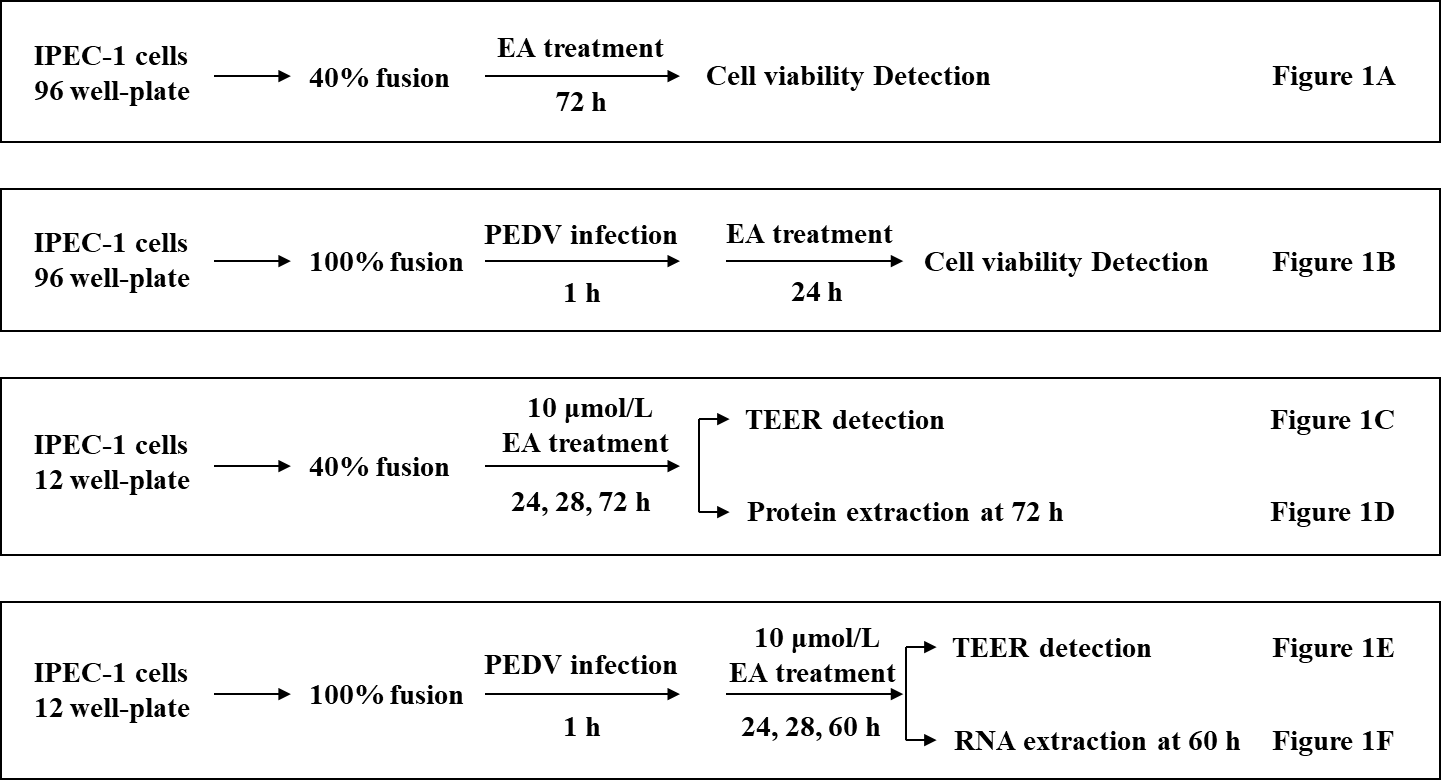


Supplemental Table 2. Primer sequences used in RT-qPCR

|  | Sequences of primers |
| --- | --- |
| *IL-6* | F: 5'-TACTGGCAGAAAACAACCTG-3' |
|  | R: 5'-GTACTAATCTGCACAGCCTC-3' |
| *IL-1β* | F: 5'-CAACGTGCAGTCTATGGAGT-3' |
|  | R: 5'-GAGGTGCTGATGTACCAGTTG-3' |
| *TNF-α* | F: 5'-TCCAATGGCAGAGTGGGTATG-3' |
|  | R: 5'-AGCTGGTTGTCTTTCAGCTTCAC-3' |
| *IL-8* | F: 5'-TTCGATGCCAGTGCATAAATA-3' |
|  | R: 5'-CTGTACAACCTTCTGCACCCA-3' |
| *CXCL2* | F: 5'-CGGAAGTCATAGCCACTCTCAA-3' |
|  | R: 5'-CAGTAGCCAGTAAGTTTCCTCCATC-3' |
| *REG3g* | F: 5'-CTGTCTCAGGTCCAAGGTGAAG-3' |
|  | R: 5'-CAAGGCATAGCAGTAGGAAGCA-3' |
| *PEDV-M* | F: 5'-TCCCGTTGATGAGGTGAT-3' |
|  | R: 5'-AGGA TGCTGAAAGCGAAAA-3' |
| *PEDV-N* | F: 5'-CGCAAAGACTGAACCCACTAACTT-3' |
|  | R: 5'-TTGCCTCTGTTGTTACTCGGGGAT-3' |
| *IFN-β* | F: 5'-AGCAGATCTTCGGCATTCTC-3' |
|  | R: 5'-GTCATCCATCTGCCCATCAA-3' |
| *MX1* | F: 5'-AGTGCGGCTGTTTACCAAG-3' |
|  | R: 5'-TTCACAAACCCTGGCAACTC-3' |
| *IFIT1* | F: 5'-GCTAAACCAAACACCGCAGAA-3' |
|  | R: 5'-GGAACTCAATCTCCTCCAAGACC-3' |
| *IFITM3* | F: 5'-CAACATCCGAAGCGAGACC-3' |
|  | R: 5'-AGTGGTGCAAACGATGATGAA-3' |
| *AQP8* | F: 5'-CTGCCTGTCGGTCATTGAGAA-3' |
|  | R: 5'-TTGAAGTGTCCGCCACTGATG-3' |
| *AQP10* | F: GGGCGTTATACTAGCCATCTAC-3' |
|  | R: CCAACTGCACCAAGGAGTAA-3' |
| *KCNJ13* | F: ATGGATGTGTCGCTGGTCTTT-3' |
|  | R: CACAACTGCTTGCCTTTACGAG-3' |
| *NHE3* | F: 5'-AAGTACGTGAAGGCCAACATCTC-3' |
|  | R: 5'-TTCTCCTTGACCTTGTTCTCGTC-3' |
| *APOA1* | F: 5'-TTTGCCACCGTGTATGTGGA-3' |
|  | R: 5'-TGTCCAGGAGTTTCAGGTTGAG-3' |
| *APOA4* | F: 5'-ACCCAGCAGCTCAACACTCTC-3' |
|  | R: 5'-GAGTCCTTGGTCAGGCGTTC-3' |
| *APOC2* | F: 5'-AGTCCGTGTTCCAGGTCCC-3' |
|  | R: 5'-CAGTGGCCTTGGCTGTATCC-3' |
| *SGLT1* | F: 5'-GGCCAGCTACCTCAAGATGC-3' |
|  | R: 5'-CAGCCCACATCATCTGGAAAC-3' |
| *RPL4* | F: 5'-GAGAAACCGTCGCCGAAT-3' |
|  | R: 5'-GCCCACCAGGAGCAAGTT-3' |


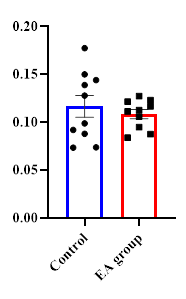


Supplemental Figure 1. Effect of EA on average daily gain of piglets. Data are presented as means ± SEMs (n = 10). Different letters indicate significant differences (*P* < 0.05) between groups. ADG, average daily gain; EA, ellagic acid.
